# Multifocal Uremic Tumoral Calcinosis From Prolonged Hyperparathyroidism in Autosomal Dominant Polycystic Kidney Disease

**DOI:** 10.7759/cureus.108102

**Published:** 2026-05-01

**Authors:** Merrick J Harris, Paola Pedraza Cruz, Shoaib Junejo

**Affiliations:** 1 Department of Medicine, The University of Toledo College of Medicine and Life Sciences, Toledo, USA

**Keywords:** autosomal dominant polycystic kidney disease (adpkd), calcium metabolism, chronic kidney disease, dialysis, end-stage renal disease, hyperparathyroidism, parathyroid disorders, parathyroidectomy, renal osteodystrophy, tumoral calcinosis

## Abstract

Secondary hyperparathyroidism (SHPT) is a common complication of chronic kidney disease. Prolonged stimulation of the parathyroid glands can lead to severe mineral bone disorders. Uremic tumoral calcinosis (UTC) is a rare but debilitating manifestation of prolonged hyperparathyroidism characterized by periarticular calcium-phosphate deposits. This case describes the progression of SHPT to multifocal UTC in a patient with autosomal dominant polycystic kidney disease (ADPKD).

A 46-year-old female with ADPKD-related end-stage renal disease (ESRD) was evaluated for renal transplantation and found to have an elevated parathyroid hormone (PTH) level. A sestamibi scan revealed a left inferior pole parathyroid adenoma, and she underwent a targeted parathyroidectomy. The remaining glands appeared normal intraoperatively and were preserved. Despite optimal medical management after parathyroidectomy, the patient's PTH levels remained markedly elevated. In the ensuing year, the patient developed complications from prolonged hyperparathyroidism, including severe bone pain, arthralgias, pathologic fractures, and multifocal UTC.

In ESRD, impaired phosphate excretion and vitamin D metabolism disrupt calcium-phosphate homeostasis, altering the feedback to the parathyroid glands. This leads to chronic elevation of PTH and promotes bone resorption, increasing the risk of pathologic fractures. In some cases, ectopic calcification may occur. Multifocal UTC reflects a severe manifestation of these systemic mineral imbalances.

This case illustrates a rare but severe complication of chronic hyperparathyroidism in the setting of ADPKD, where refractory SHPT progressed to multifocal UTC despite surgical and medical interventions. Early recognition and aggressive, multidisciplinary management are critical to mitigate the progression of this condition and preserve quality of life.

## Introduction

Secondary hyperparathyroidism (SHPT) develops in patients with chronic kidney disease (CKD) due to increased phosphate retention, reduced 1-alpha hydroxylase activity, and vitamin D deficiency, resulting in hypocalcemia and compensatory parathyroid hormone (PTH) secretion [1]. Chronic stimulation of the parathyroid glands causes parathyroid hyperplasia and eventual autonomous PTH production, leading to tertiary hyperparathyroidism. Due to PTH-stimulated bone resorption and subsequent calcium release, prolonged hyperparathyroid states can cause significant morbidity in patients, including debilitating arthralgias, renal osteodystrophy, pathologic fractures, and, in severe cases, tumoral calcinosis [2]. Uremic tumoral calcinosis (UTC) is a rare but debilitating manifestation of CKD characterized by periarticular, multilobulated calcium-phosphate deposits, most often affecting the hip, shoulder, and pelvic girdle [3]. This case highlights the clinical course and management of a patient with significant complications progressing to multifocal UTC from prolonged hyperparathyroidism secondary to autosomal dominant polycystic kidney disease (ADPKD).

## Case presentation

A 46-year-old female with a past medical history of hypertension, hyperlipidemia, and ADPKD with a five-year history of end-stage renal disease (ESRD) on nightly cycles of peritoneal dialysis was found to have an elevated PTH level of 2681 pg/mL with a normal calcium level after undergoing renal transplant evaluation (Table 1).

**Table 1:** Lab results from the blood sample drawn during pre-renal transplantation evaluation.

Investigation	Patient's value	Reference range
Parathyroid hormone	2681 pg/mL	15-65 pg/mL
Serum calcium	9.4 mg/dL	8.5-10.2 mg/dL
Ionized calcium	4.76 mg/dL	4.65-5.28 mg/dL
Phosphate	8.9 mg/dL	2.7-4.5 mg/dL
Alkaline phosphatase	201 U/L	38-126 U/L

These findings were consistent with chronic SHPT despite ongoing management with calcitriol, calcium supplements, cinacalcet, and phosphate-binding medications. The patient’s lab values from six months prior to this evaluation are included in Table 2.

**Table 2:** Lab results six months prior to pre-renal transplantation evaluation.

Investigation	Patient's value	Reference range
Parathyroid hormone	2334 pg/mL	15-65 pg/mL
Serum calcium	9.9 mg/dL	8.5-10.2 mg/dL
Ionized calcium	4.80 mg/dL	4.65-5.28 mg/dL
Phosphate	8.2 mg/dL	2.7-4.5 mg/dL
Alkaline phosphatase	170 U/L	38-126 U/L

Due to the markedly elevated PTH levels, a sestamibi scan was performed, which demonstrated a left inferior pole parathyroid adenoma. Subsequently, the patient underwent a targeted left inferior pole parathyroidectomy. Intraoperatively, the patient’s PTH level dropped to 1700 pg/mL after the selected gland was excised, and the remaining parathyroid glands appeared normal and were not removed. Surgical pathology of the excised gland demonstrated 2.02 grams of enlarged, hypercellular parathyroid tissue that was diagnosed as a parathyroid adenoma. Postoperatively, the patient’s course was complicated by dysphonia, prolonged neck pain, and wound dehiscence several months later. Despite surgical intervention, the patient’s PTH levels continued to remain elevated over 1000 pg/mL, and she began an optimized medical regimen for SHPT with cinacalcet, calcitriol, ergocalciferol, and sucroferric oxyhydroxide.

**Figure 1:**
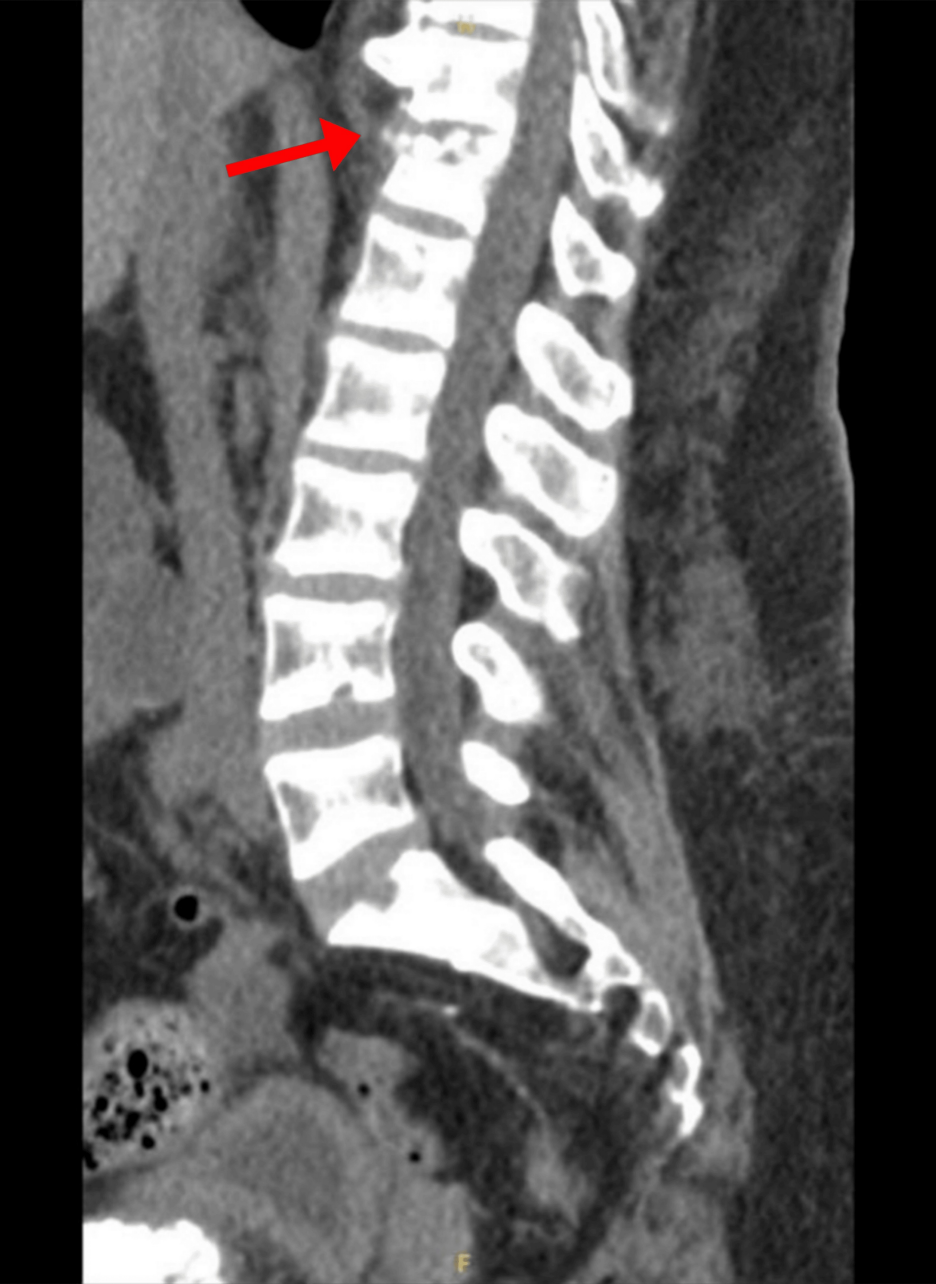
CT of the lumbar spine demonstrates T12 superior endplate compression fracture and “rugger jersey spine” appearance due to vertebral bone resorption from chronic hyperparathyroidism.

Despite this medical therapy, the patient continued to experience significant complications from hyperparathyroidism, which led to multiple emergency department visits. Five months after undergoing targeted parathyroidectomy, the patient presented to the ED with intractable abdominal pain and polyarthralgia. Labs revealed an elevated PTH once again, and computed tomography (CT) of the lumbar spine revealed excess osteoid deposition and increased bone resorption, termed “rugger jersey spine,” with an acute compression fracture of the T12 superior endplate (Figure 1). These findings were consistent with sequelae of chronic, uncontrolled hyperparathyroidism.

**Figure 2:**
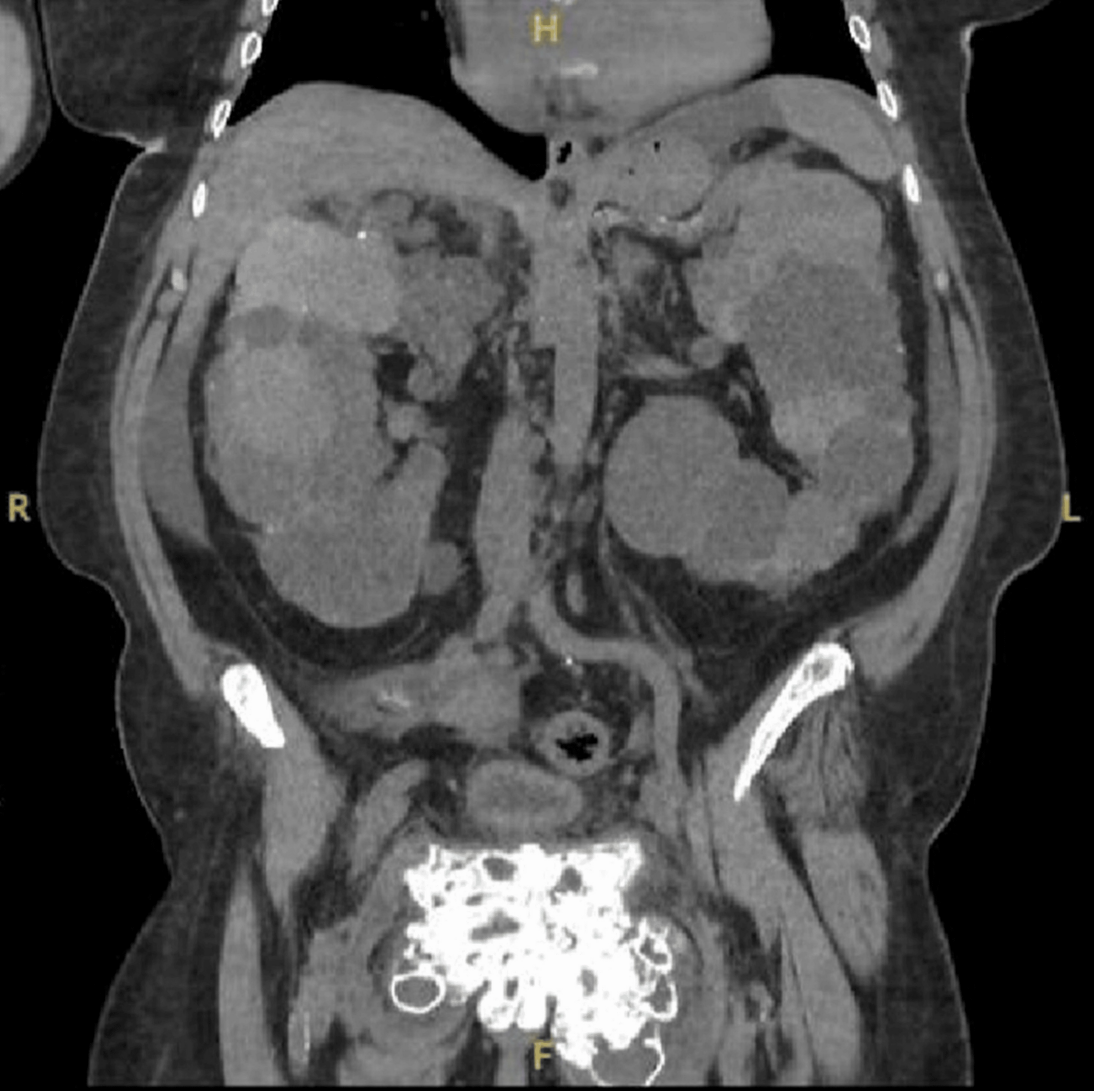
CT coronal view of the abdomen and pelvis demonstrates significant uremic tumoral calcinosis of the pubic symphysis, and polycystic kidneys are also shown.

**Figure 3:**
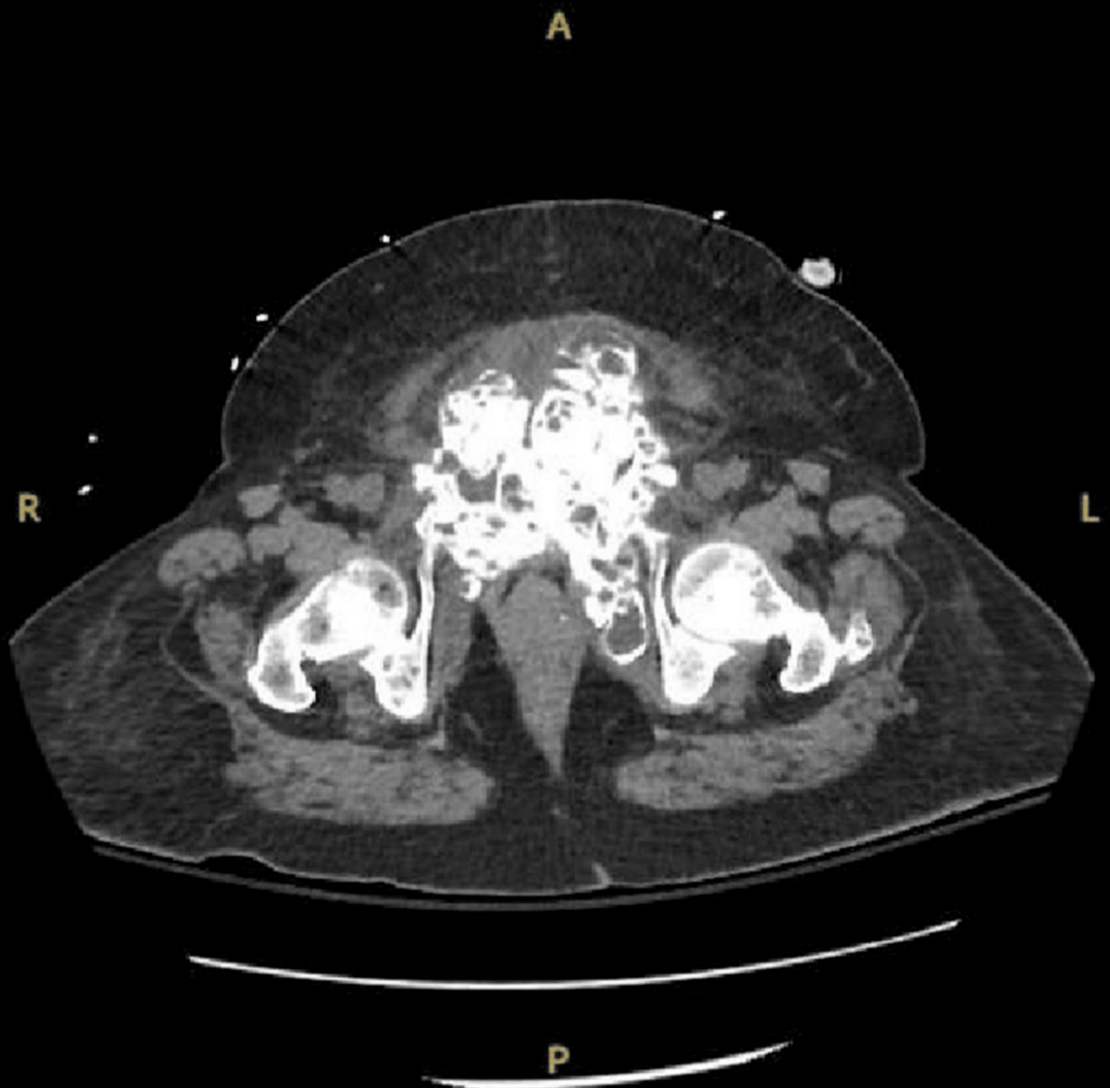
CT axial view of the abdomen and pelvis redemonstrates uremic tumoral calcinosis of the pubic symphysis, with the left side worse than the right in posterior extension.

**Figure 4:**
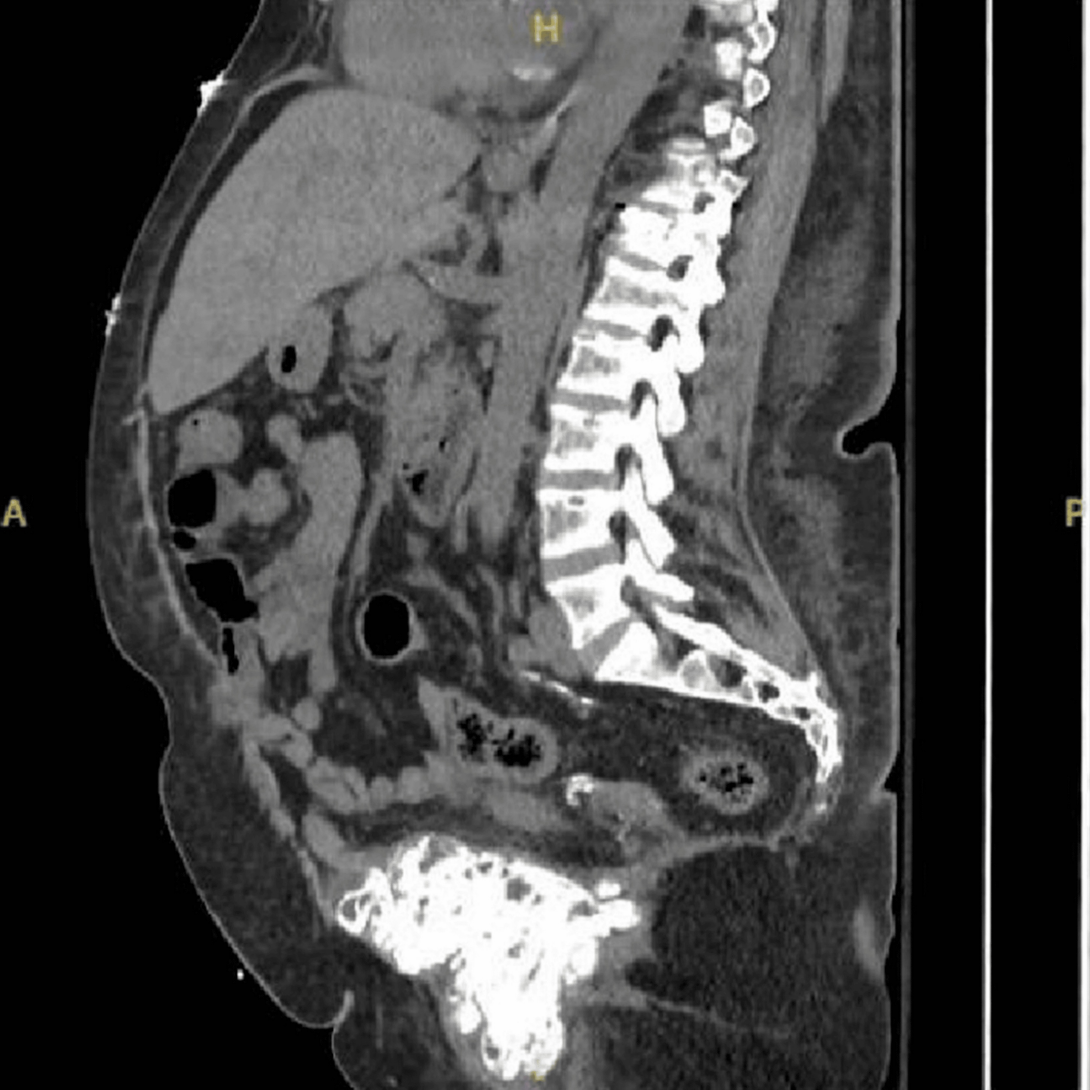
CT sagittal view of the abdomen and pelvis. Sagittal view of pubic uremic tumoral calcinosis, with the left worse than the right and a vertical measurement of 62.5 mm. Lytic changes of the spine and T12 fracture were also re-demonstrated as a result of renal osteodystrophy.

At this time, due to the patient experiencing severe abdominal pain, a CT of the abdomen and pelvis was also obtained. This demonstrated significant UTC around the pubic symphysis (Figures 2-4), which was re-demonstrated on follow-up imaging.

**Figure 5:**
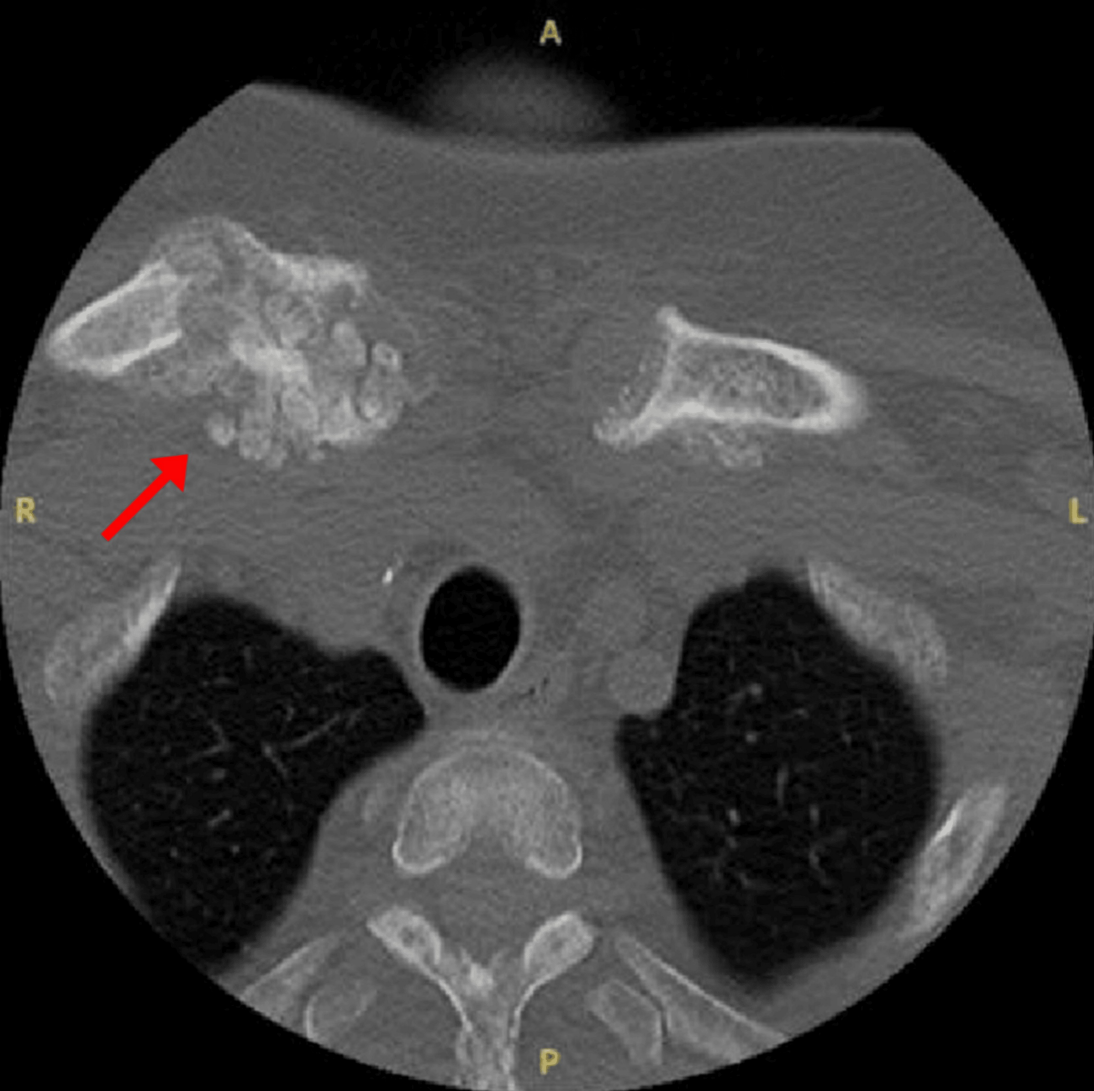
CT axial view of the sternoclavicular region.

**Figure 6:**
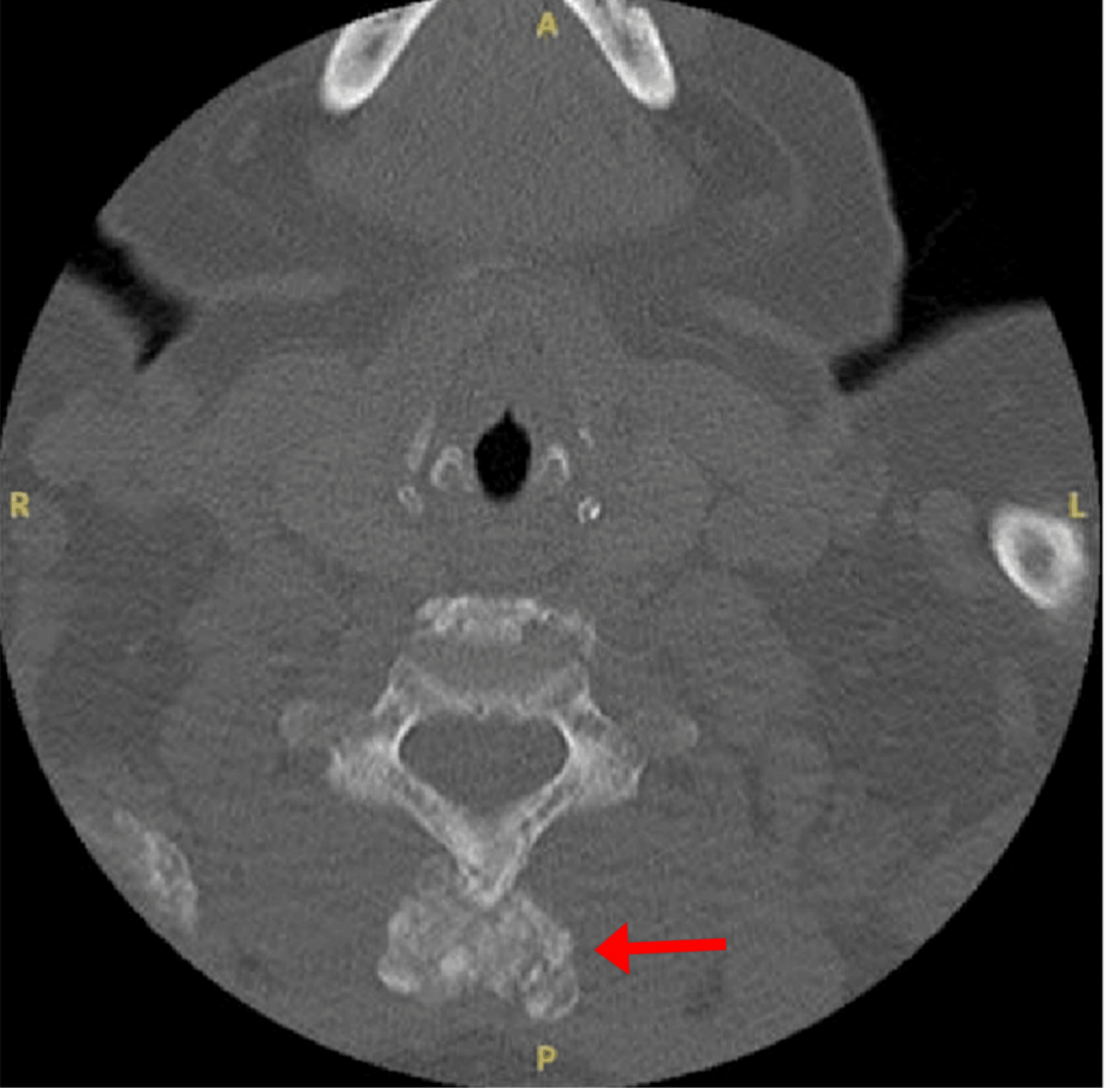
CT axial view demonstrating spinous process involvement.

**Figure 7:**
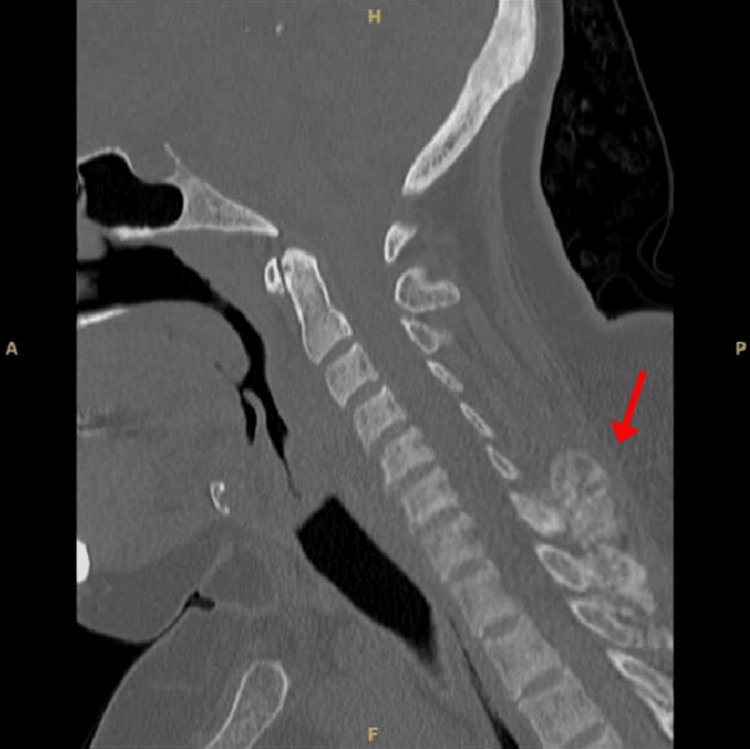
CT sagittal view of the spinous process involvement.

Three months later, the patient presented to the ED with acute back and pelvic pain. Repeat imaging revealed worsening of the T12 fracture and pubic symphysis UTC. Ten months after her initial subtotal parathyroidectomy, the patient presented to the ED again with right shoulder pain, and an X-ray revealed a nondisplaced fracture of the acromial end of the right clavicle and osteopenia along with numerous sclerotic bony lesions. CT of the cervical spine revealed spinous process remodeling along with bilateral sternoclavicular UTC (Figures 5-7).

**Figure 8:**
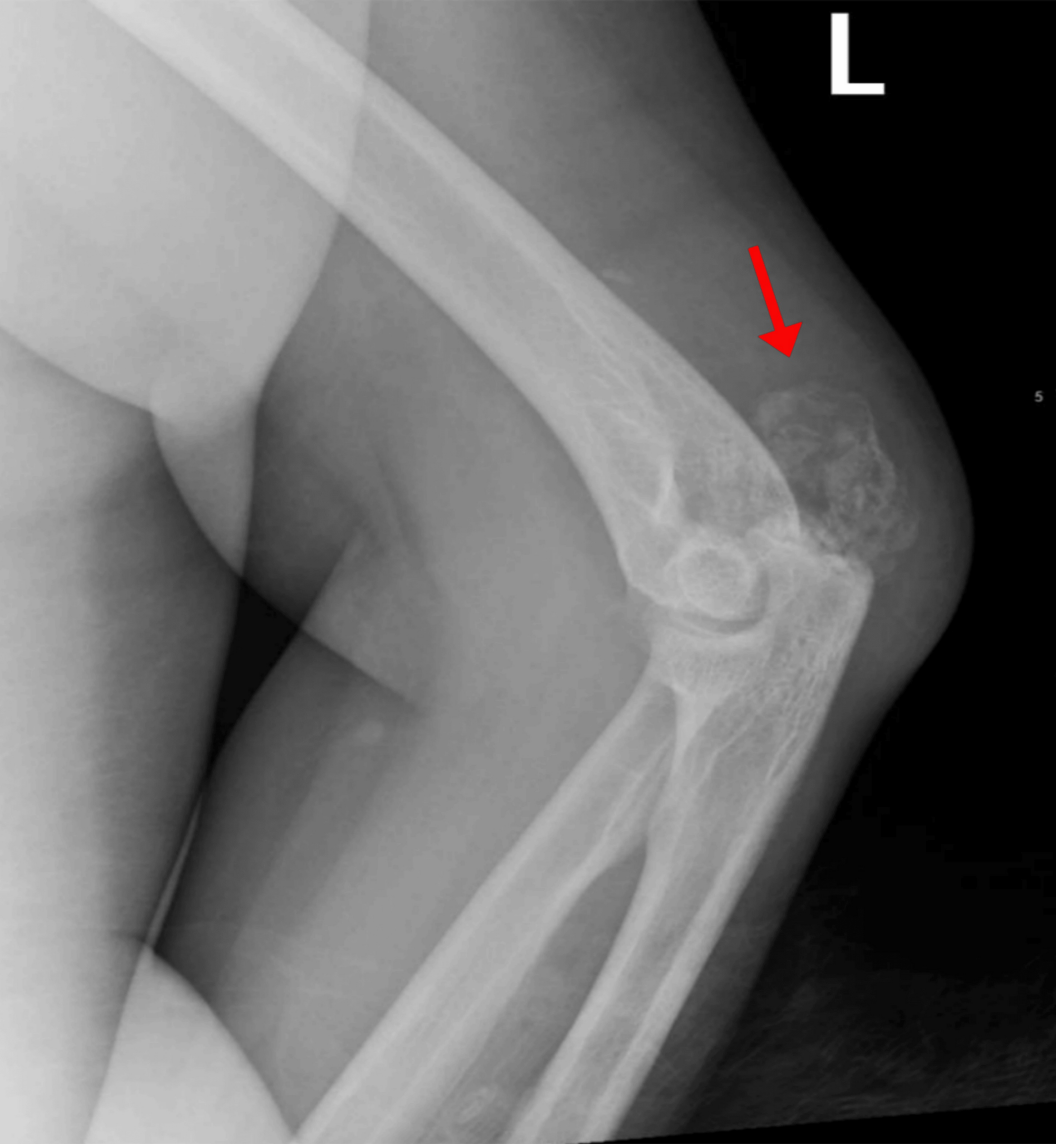
X-ray of the left elbow demonstrating periarticular calcium-phosphate deposits.

**Figure 9:**
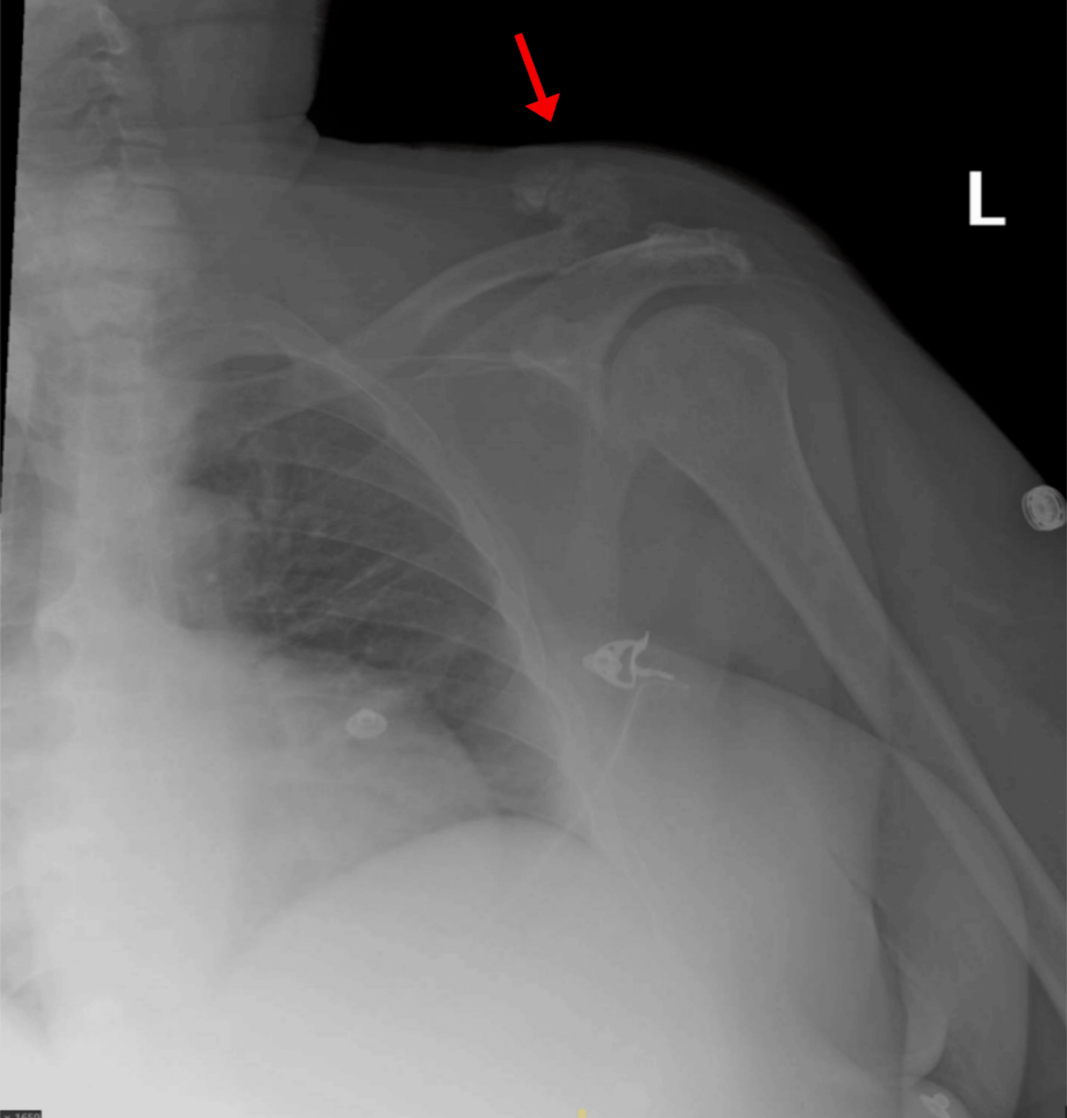
X-ray demonstrating left acromioclavicular joint involvement.

Over the next four months, the patient visited the ED seven more times due to polyarthralgia, bone pain, and abdominal pain with no inciting traumatic events. Imaging consistently displayed progressive UTC, with new soft tissue deposits discovered around the left elbow and left acromioclavicular joints (Figures 8, 9). The patient was referred to orthopedic oncology for further management of the progressive, multifocal UTC.

Currently, the patient is still awaiting renal transplantation and is being managed on a combination of lanthanum carbonate, sevelamer carbonate, and sucroferric oxyhydroxide for phosphate-binding therapy, as well as calcitriol, cinacalcet, and allopurinol. Pain is controlled with a combination of oral narcotics, gabapentin, and lidocaine patches. The patient’s mobility is limited, but the patient ambulates with assistive devices, functions independently, and can perform all activities of daily living.

## Discussion

PTH plays a critical role in calcium and phosphate homeostasis by increasing distal tubular calcium reabsorption, enhancing phosphate excretion, and stimulating production of active vitamin D, which results in increased intestinal absorption of calcium and phosphate [4]. In CKD, the kidneys fail to respond adequately to PTH, disrupting this regulatory loop and leading to aberrant PTH release and SHPT [5]. Patients with ADPKD specifically develop hyperparathyroidism at a high frequency due to the disease’s progressive cystic destruction of renal tissue. As this inherited condition progresses, cysts replace healthy renal parenchyma, decreasing the number of functional nephrons, leading to mineral imbalances and CKD, triggering classic SHPT [6]. Patients with ADPKD who require long-term dialysis are particularly susceptible to complications from prolonged exposure to abnormal calcium and phosphate homeostasis, with a 37% higher risk of fracture and markedly increased skeletal fragility than patients with kidney disease of different etiologies [6].

Our patient’s course illustrates this pathophysiology, with excessive PTH release causing extensive bone resorption and renal osteodystrophy, significant bone pain, arthralgias, frequent emergency department visits, and the development of multifocal uremic tumoral calcinosis. The manifestation of multifocal UTC is a rare but severe sequela of poorly controlled CKD. UTC results from the precipitation of calcium-phosphate complexes in soft tissues. The pathogenesis is multifactorial and involves an interplay of chronic hyperphosphatemia, elevated PTH, local tissue injury, and altered bone turnover [7]. In our patient, UTC manifested as extensive periarticular and axial skeletal deposits that involved the pelvis, spine, and sternoclavicular joints, as well as soft tissue calcifications that led to significant pain and functional impairment. Management of advanced CKD, especially secondary to ADPKD, and concomitant UTC requires a multidisciplinary approach. Medical therapy focuses on controlling calcium and phosphate levels, typically with phosphate binders, vitamin D analogs to suppress PTH, calcimimetics, and dialysis. Surgical parathyroidectomy remains standard for refractory cases with subtotal or total resection depending on the extent of disease [8]. However, there are important surgical and postoperative considerations that can present a challenge to patients, such as dysphonia, delayed healing, and other surgical wound concerns [9]. These complications, combined with the progressive skeletal fragility and recurrent fractures observed in our patient, reflect the systemic burden and morbidity associated with ESRD complicated by UTC.

This case highlights the clinical complexity of hyperparathyroidism secondary to ESRD from ADPKD, especially in the setting of UTC. This patient’s persistently elevated PTH levels after surgical excision of a supposed parathyroid adenoma suggest a transition from SHPT to tertiary hyperparathyroidism. In this case, only one parathyroid gland was excised due to the other glands appearing grossly normal intraoperatively, but perhaps if multiple glands had been sampled and examined histopathologically, the diagnosis of tertiary hyperparathyroidism would have been confirmed early on.

It demonstrates the need for early, aggressive control of CKD to prevent irreversible skeletal and soft tissue damage, and the importance of a multidisciplinary approach to improve outcomes and optimize the quality of life of individuals affected by this condition.

## Conclusions

This case illustrates how ADPKD can induce a state of prolonged hyperparathyroidism leading to severe metabolic derangements, bone deterioration, and multifocal UTC. Early and aggressive management, including multidisciplinary surgical and medical therapies, is essential to prevent irreversible complications.
